# Application of Artificial Intelligence in Breast Ultrasound Diagnosis

**DOI:** 10.3390/diagnostics16121839

**Published:** 2026-06-14

**Authors:** Jian Zhang, André Pfob, Eva Reisig, Lie Cai

**Affiliations:** 1 Faculty of Medicine, Freiburg University, 79085 Freiburg, Germany; jian.zhang@email.uni-freiburg.de; 2 Department of Breast Unit, Heidelberg University Hospital, 69120 Heidelberg, Germany; andre.pfob@kse-hd.de (A.P.); eva.reisig@stud.uni-heidelberg.de (E.R.); 3 Department of Breast Center, Hospital St Elisabeth, 69121 Heidelberg, Germany

**Keywords:** artificial intelligence, ultrasound diagnosis, breast ultrasound, BI-RADS, computer-aided diagnosis, deep learning, radiomics, elastography, automated breast ultrasound, axillary lymph node

## Abstract

Artificial intelligence (AI) is reshaping ultrasound diagnosis by converting operator-dependent grayscale, Doppler, elastography, contrast-enhanced, automated-volume, and video data into reproducible decision support. In breast ultrasound, the most mature evidence involves benign–malignant lesion classification, BI-RADS risk stratification, reduction in unnecessary biopsy in selected low-risk lesions, assistance for less experienced readers, automated breast volume scanning, video-based assessment, axillary staging, and prediction of biologic markers such as molecular subtype, HER2 status, Ki-67 expression, lymphovascular invasion, and nodal metastasis. AI does not replace sonographers, radiologists, pathologists, or clinical judgment; rather, it can standardize feature extraction, prompt second-reader review, quantify uncertainty, and integrate imaging with clinical context. This review summarizes current clinical applications of AI in ultrasound diagnosis, which has a strong recent multicenter evidence base. It also discusses implementation requirements, including standardized acquisition, external validation, calibration, imaging–pathology concordance, workflow integration, data security, and equity across scanners and patient populations.

## 1. Introduction: From Ultrasound Imaging to AI-Assisted Diagnosis

AI-assisted ultrasound diagnosis builds on one of the most frequently used imaging methods in breast care because it is portable, widely available, radiation-free, repeatable, and capable of real-time correlation with focal symptoms, mammographic abnormalities, surgical scars, palpable lumps, skin changes, or axillary findings. These advantages explain why ultrasound is embedded in diagnostic pathways, even in health systems where mammography remains the backbone of population screening. The current ACR BI-RADS framework continues to emphasize standardized ultrasound lexicon, final assessment, and management linkage, while recent practice parameters describe diagnostic breast ultrasound and whole-breast ultrasound as structured examinations rather than informal scanning exercises [1,2,3]. In symptomatic care, the ACR Appropriateness Criteria for palpable breast masses still place ultrasound at the center of age-adapted evaluation, particularly for younger patients and for targeted assessment after mammography or tomosynthesis in older patients [4]. In screening and risk-adapted care, supplemental ultrasound is considered in selected women with dense breasts or elevated risk, with the key trade-off that additional cancers may be found at the cost of more false-positive findings and biopsies [1].

The rationale for applying AI to ultrasound is strongest where human interpretation is variable, image acquisition is dynamic, and the diagnostic threshold has direct clinical consequences. Conventional ultrasound remains essential because it is real-time, inexpensive, radiation-free, and directly linked to biopsy guidance. Its weakness is that image quality and interpretation vary with operator skill, scanner settings, lesion selection, and local reporting culture. AI methods, including machine learning, deep learning, radiomics, and multimodal fusion models, attempt to reduce this variability by learning reproducible image patterns and combining them with clinical factors.

This narrative review, therefore, shifts the focus from ultrasound as a modality to AI as a diagnostic layer built on ultrasound data. Breast ultrasound is used as the main clinical model, while lessons are framed more broadly for AI-assisted ultrasound diagnosis. The evidence base includes prospective and multicenter studies of ultrasound computer-aided diagnosis, BI-RADS decision support, shear-wave elastography, Doppler flow imaging, contrast-enhanced ultrasound, automated breast volume scanning, freehand ultrasound video, axillary assessment, and imaging-based tumor phenotyping.

The central theme is that AI in ultrasound diagnosis should be task-specific. A model for distinguishing benign from malignant masses is not automatically suitable for whole-breast screening, axillary staging, treatment response assessment, or molecular prediction. Clinical value depends not only on the area under the curve but also on sensitivity, false-negative rate, benign biopsy reduction, reader workload, calibration, interpretability, and whether the output changes management safely.

## 2. Literature Search Strategy and Selection Criteria

A comprehensive literature search was conducted to identify relevant studies investigating the application of artificial intelligence (AI), machine learning (ML), deep learning (DL), and radiomics in breast ultrasound imaging. The primary database queried was PubMed/MEDLINE. The search strategy employed a combination of Medical Subject Headings (MeSH) and free-text keywords to capture a broad spectrum of relevant literature. The Boolean search string was constructed as follows:

((“breast cancer”[Title/Abstract] OR “breast neoplasm”[Title/Abstract] OR “breast tumor*”[Title/Abstract] OR “breast carcinoma”[Title/Abstract] OR “mammary cancer”[Title/Abstract]) AND (“artificial intelligence”[Title/Abstract] OR “machine learning”[Title/Abstract] OR “deep learning”[Title/Abstract] OR “neural network*”[Title/Abstract] OR “convolutional neural network*”[Title/Abstract] OR CNN[Title/Abstract] OR transformer*[Title/Abstract] OR radiomics[Title/Abstract] OR “computer-aided”[Title/Abstract] OR CAD[Title/Abstract])).

To ensure the review reflects the most contemporary advancements in this rapidly evolving field, a temporal filter was applied to restrict the publication date from January 2016 to May 2026.

The inclusion criteria were strictly defined as follows: (1) original research articles evaluating AI or ML algorithms applied to breast ultrasound (including B-mode, shear wave elastography, contrast-enhanced ultrasound, and automated breast ultrasound); (2) studies reporting quantitative diagnostic, prognostic, or predictive performance metrics (e.g., Area Under the Curve [AUC], sensitivity, specificity, or negative predictive value); and (3) articles published in peer-reviewed journals in the English language.

Conversely, the exclusion criteria encompassed: (1) review articles, meta-analyses, case reports, editorials, and conference abstracts; (2) studies focusing solely on other imaging modalities (such as MRI or standalone mammography) without a primary ultrasound component; (3) non-human (in vitro or animal) studies; and (4) studies lacking sufficient technical validation or clear clinical endpoints.

**Figure 1:**
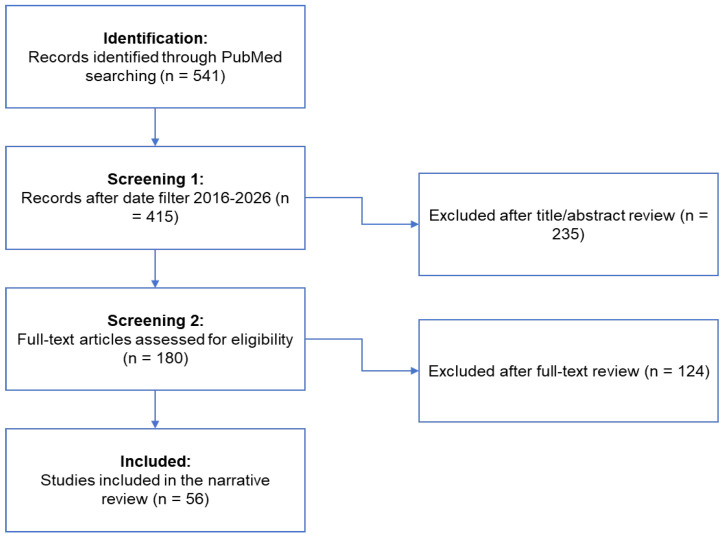
PRISMA flowchart of the literature search and study selection process for AI applications in breast ultrasound.

The remaining 180 articles underwent a rigorous full-text review. In this phase, an additional 124 articles were excluded due to insufficient performance data, lack of independent test cohorts, or poor methodological quality. Ultimately, 56 core studies were deemed highly relevant and included in this narrative review (Figure 1).

## 3. Diagnostic Tasks Addressed by AI in Ultrasound

AI-assisted ultrasound begins with the same clinical questions that guide human scanning: Is there a true lesion? Is it cystic or solid? Does it meet BI-RADS criteria for follow-up or biopsy? Is there a sonographic correlate for a mammographic, tomosynthesis, or MRI finding? Can the axilla be staged noninvasively? Can imaging features enrich the prediction of tumor biology or response? AI adds value when it provides a reproducible probability estimate, highlights overlooked features, reduces interobserver variability, or supports triage in settings where breast imaging expertise is limited.

The first recurring question is whether a finding is a true lesion and, if so, whether it is cystic, solid, inflammatory, traumatic, postoperative, or suspicious for malignancy. Simple cysts, complicated cysts, abscesses, fat necrosis, fibroadenomas, papillomas, phyllodes tumors, granulomatous mastitis, and invasive cancers can overlap clinically. Ultrasound narrows this differential by showing internal echoes, margins, posterior features, orientation, ductal extension, architectural distortion, skin involvement, and vascularity. Radiomics studies highlight that some clinically confusing entities, such as granulomatous lobular mastitis and invasive breast cancer, can share conventional features but may still contain quantifiable sonographic patterns that improve discrimination [5]. Similarly, triple-negative breast cancers may mimic fibroadenomas with apparently benign morphology, a reminder that morphology must be interpreted alongside patient age, growth, symptoms, and context [6].

The second question is whether a lesion requires biopsy, short-interval follow-up, or routine care. BI-RADS creates the management language for that decision. Ultrasound is especially influential in BI-RADS 3 and BI-RADS 4A categories, where the clinical stakes are substantial: overcalling creates anxiety and unnecessary biopsy, while undercalling can delay cancer diagnosis. AI and CAD studies have therefore concentrated on low-suspicion lesions because this is where decision support may have the largest clinical yield. Prospective and multicenter studies of ultrasound CAD and decision support suggest that assistance can improve reader performance, particularly for less experienced radiologists, but the safest use is as a second reader or structured prompt rather than an autonomous final assessor [7,8,9,10,11,12,13]. Studies specifically targeting BI-RADS 4A lesions show that deep learning may help refine malignancy probability and reduce unnecessary intervention, but any downgrade strategy must preserve sensitivity and be audited locally [14].

The third question is whether ultrasound can add value when mammography is limited by breast density or when resources are constrained. Supplemental ultrasound can detect mammographically occult cancers in dense breasts, but the effect depends on baseline risk, screening interval, local expertise, and tolerance for false positives. In low-resource settings, ultrasound triage supported by AI has been studied as a way to prioritize women with palpable lumps when access to full diagnostic imaging is limited [13]. This use should not be confused with replacing established screening programs, but it illustrates the distinctive strength of ultrasound: it can bring diagnostic imaging close to the point of care.

The fourth question is whether ultrasound can support staging and treatment planning. Axillary ultrasound is a routine part of many breast cancer pathways, and recent ultrasound radiomics, elastography, and deep learning models aim to improve the prediction of axillary lymph node metastasis, lymphovascular invasion, and residual nodal disease after neoadjuvant therapy [15,16,17,18,19]. Other studies explore the prediction of molecular subtype, HER2-low status, Ki-67 expression, and luminal versus non-luminal biology from ultrasound or ultrasound–pathology fusion [20,21,22,23,24,25,26]. These major clinical applications of AI in ultrasound diagnosis are summarized in Table 1. Their near-term role is to support triage, sampling strategy, multidisciplinary planning, and risk communication.

## 4. Data, Model, and Interpretive Foundations

The diagnostic reliability of AI-assisted ultrasound begins with technique and data quality. High-frequency linear transducers, appropriate focal-zone placement, optimized gain, harmonic imaging when useful, consistent compression, and imaging in radial and antiradial or orthogonal planes are not cosmetic details. They determine whether margins are visible, whether a lesion is truly taller-than-wide, whether posterior features are real, and whether internal vascularity or elasticity can be interpreted. ACR practice parameters emphasize that diagnostic breast ultrasound should be documented with lesion location, size in three dimensions when appropriate, distance from nipple, representative images, and correlation with other imaging and clinical findings [2]. The 2025 version of the BI-RADS framework continues the same principle: ultrasound findings should be reported in a lexicon that links morphology to management rather than in free-text impressions that cannot be audited [27].

B-mode morphology remains the starting point for AI-assisted ultrasound. Irregular shape, noncircumscribed margins, nonparallel orientation, posterior shadowing, echogenic halo, ductal extension, architectural distortion, skin change, and suspicious lymph nodes are still clinically meaningful. AI should not obscure these descriptors; the best systems make them more consistent, quantify them more reproducibly, or combine them with features that are difficult for humans to perceive.

The diagnostic output must also remain tied to management. A low model score is useful only if it can safely support routine care or follow-up; a high score is useful only if it supports biopsy, additional imaging, or multidisciplinary planning. AI should therefore be evaluated in relation to BI-RADS categories, pathology, follow-up, and downstream decisions rather than as an isolated image-classification exercise.

Operator dependence remains the central limitation. Unlike mammography, ultrasound image quality and completeness depend heavily on the person holding the probe. Hand pressure can change lesion shape, Doppler signal can be lost with compression, and incomplete scanning can miss a subtle finding. Automated breast ultrasound was developed partly to reduce this dependence by acquiring standardized volumetric data that can be reviewed after acquisition [28,29]. Video-based and whole-lesion-aware deep learning approaches similarly attempt to capture more of the dynamic scanning information rather than relying on one or two static frames [30,31]. These methods are appealing because a breast mass is a three-dimensional structure, but they require storage, workflow, annotation, and quality control systems that many clinics are still building.

## 5. AI-Assisted B-Mode Ultrasound, BI-RADS, and Lesion Classification

For AI in ultrasound diagnosis, the most mature task is benign–malignant classification of a mass. Recent studies show strong interest in decision support for this task because it affects biopsy rates directly. A prospective multicenter study of ultrasound CAD for distinguishing breast masses demonstrated that CAD systems can provide clinically relevant discrimination when evaluated across centers [7]. AI-based BI-RADS categorization has also been studied as a way to reduce excessive follow-ups and biopsies while maintaining high negative predictive value [8]. In readers without breast ultrasound expertise, deep learning-based CAD improved lesion classification in a prospective multicenter setting, suggesting possible value for hospitals where specialized breast imaging expertise is scarce [9]. Similar benefits have been reported for decision-support systems used by radiologists with different experience levels and for S-detect assistance, particularly among less experienced readers [11,12].

The main clinical opportunity for AI is to reduce variation around difficult thresholds. BI-RADS 4A is a good example. These lesions have low but non-negligible malignancy probability, and the default recommendation is biopsy. A deep learning model designed for BI-RADS 4A ultrasound lesions reported high diagnostic performance and suggested that selected lesions could be reclassified in a way that changes clinical management [14]. The promise is fewer unnecessary biopsies, lower patient anxiety, and lower cost. The risk is a false reassurance pathway in which a cancer is downgraded because a model overfits local data, scanner settings, or lesion selection. Clinical adoption should therefore require prospective testing, transparent sensitivity targets, local audit of false negatives, and explicit rules for when clinical concern overrides the model.

AI radiomics and transfer learning studies highlight the value of feature fusion. Conventional radiomics quantifies shape, intensity, texture, and wavelet features; transfer learning extracts deep features from pretrained neural networks; clinical models add age, symptoms, lesion size, and other patient factors. In a multicenter retrospective study, fusion of conventional radiomics and transfer-learning features achieved higher performance than either approach alone [32]. Another multicenter breast ultrasound radiomics study supported clinical value for differential diagnosis of benign and malignant lesions [33,34]. These results are consistent with a broader principle: ultrasound diagnosis is strongest when morphology, quantitative image features, and clinical context are integrated rather than isolated.

AI decision support is also being tested in difficult differential diagnoses. Granulomatous lobular mastitis can mimic invasive breast cancer, leading to unnecessary surgery or delayed inflammatory treatment. A sonogram-radiomics model combining ultrasound features and radiomics improved discrimination between granulomatous mastitis and invasive breast cancer across multicenter data [5]. Triple-negative breast cancer can mimic fibroadenoma, and ultrasound texture radiomics has been explored as a way to distinguish aggressive malignancy from benign circumscribed lesions [6]. Phyllodes tumors are another challenge because they overlap with fibroadenomas and vary from benign to malignant; a 2025 multicenter study explored hierarchical diagnosis using deep learning of ultrasound images [35]. These studies remind clinicians that “benign-looking” does not mean “benign” when the clinical context is discordant.

## 6. Multiparametric Ultrasound as Input for AI Models

Elastography adds a mechanical dimension that AI models can combine with morphology. Malignant lesions are often stiffer than benign lesions because of cellularity, desmoplasia, and tissue invasion, although stiffness can also occur with fibrosis, inflammation, or prior treatment. Strain elastography and shear-wave elastography can improve specificity when used alongside B-mode ultrasound. Early feasibility work combining ultrasound elastography CAD with BI-RADS lexicon showed improved areas under the receiver operating characteristic curve and better interobserver agreement [36]. For instance, the INSPiRED 002 study evaluated multimodal shear-wave elastography in an international cohort, demonstrating that biopsies for benign lesions could be reduced while maintaining sensitivity in the external validation set [37]. More recently, the INSPiRED 006 study developed a deep learning model for breast shear-wave elastography, illustrating the transition from simple stiffness thresholds to AI-assisted multiparametric interpretation [38].

Elastography is also being evaluated beyond primary mass diagnosis. A radiomics model based on shear-wave elastography assessed axillary lymph node status in early-stage breast cancer [15]. Another prospective multicenter diagnostic study suggested that elastography-based AI could help predict axillary status after neoadjuvant chemotherapy in patients initially presenting with nodal involvement [16]. These studies are clinically attractive because axillary surgery has become more selective. If imaging could more accurately identify residual nodal disease or nodal clearance after therapy, it might improve surgical planning and reduce overtreatment. At the same time, the axilla remains a difficult target: nodal micrometastases, therapy-related fibrosis, and sampling error can defeat both conventional imaging and AI models. Pathologic confirmation remains necessary when imaging results would alter treatment.

Doppler ultrasound contributes vascular information. Malignant tumors often show internal vascularity, penetrating vessels, or chaotic vascular architecture, but Doppler is technically sensitive to compression, motion, and machine settings. Deep learning applied to two-dimensional color Doppler flow imaging improved classification of breast masses in a multicenter study, suggesting that vascular pattern may add discriminative information when analyzed systematically [17]. In routine practice, Doppler is best treated as supportive evidence rather than a decisive single sign.

Contrast-enhanced ultrasound and super-resolution ultrasound push vascular assessment further. CEUS can show enhancement kinetics and microvascular perfusion, and a 2025 multicenter study developed a CEUS radiomics model for predicting axillary lymph node metastasis and prognosis in breast cancer [39]. Super-resolution ultrasound radiomics has been explored for predicting upstaging of ductal carcinoma in situ, a clinically important problem because DCIS diagnosed on biopsy may conceal invasive disease [40]. These techniques are promising but remain less widely available than B-mode ultrasound and elastography. Their clinical value will depend on standardized acquisition, contrast safety workflows, reproducible radiomics extraction, and evidence that the result changes management rather than simply improving an AUC.

## 7. Automated Breast Ultrasound, Ultrasound Video, and AI

Automated breast ultrasound and ultrasound video provide richer AI inputs and represent a shift from selected still images toward more complete lesion or whole-breast representation. Automated 3D breast ultrasound can acquire standardized volumes, reduce dependence on the individual operator, and allow coronal plane review. A concept study validating radiologists’ findings with CAD software in automated 3D breast ultrasound showed the implementation challenges and potential of AI in this setting [28]. A 2025 multicenter diagnostic study using automated breast volume scanner images reported deep learning-based diagnosis of breast lesions, reflecting a maturing evidence base for volumetric ultrasound analysis [29].

Video-based AI has a different logic. A static image may capture a favorable or unfavorable slice, while a video sweep contains contextual information about lesion boundaries, surrounding tissue, probe movement, and whole-lesion heterogeneity. A retrospective multicenter study evaluated deep learning in video-based ultrasonography for breast cancer diagnosis [31], and a prospective multicenter study developed a whole-lesion-aware network based on freehand ultrasound video [30]. These studies are especially relevant because they attempt to model how human experts actually scan: not by judging one frame in isolation, but by building a mental three-dimensional impression. The challenge is that videos are large, annotations are labor-intensive, and acquisition protocols vary. For clinical implementation, video AI will need robust quality checks, frame selection transparency, and clear workflow design so that it saves time rather than creating a second reading burden.

Whole-breast ultrasound for screening and staging has its own niche. Practice guidance frames it as a structured examination, not merely a long targeted scan [27]. In dense-breast supplemental screening, automated or handheld whole-breast ultrasound may detect additional cancers but produces recalls, benign biopsies, and follow-up exams [27]. In staging, whole-breast ultrasound may detect multifocal or multicentric disease, but MRI is often more sensitive. The practical decision should be based on risk, breast density, local expertise, access, and whether the result will alter surgery or systemic treatment.

## 8. Diagnostic Performance and Study Quality

Recent studies show several converging directions. First, multicenter data are increasingly used to test whether AI and radiomics survive variation in scanner vendor, acquisition protocol, reader expertise, and patient population. The specific study designs, US vendor diversity, and headline performances of representative recent multicenter studies are detailed in Table 2. Second, many studies evaluate reader assistance rather than autonomous diagnosis, which is closer to clinical deployment. Third, AI ultrasound research is expanding from binary lesion classification to axillary staging, biopsy reduction, biologic prediction, and risk-adapted triage.

## 9. Screening, Risk Stratification, and Multimodal AI Diagnosis

AI-assisted ultrasound is not a universal substitute for mammography, but it is a powerful complement. The Oslo Tomosynthesis Screening Trial showed the importance of digital breast tomosynthesis in screening, reinforcing that X-ray-based modalities remain central for population detection and calcifications [42]. Mammography and DBT AI studies have shown strong performance for cancer detection, recall triage, false-negative detection, and prospective screen-reading support [41,43,44,45,46,47,48]. These studies matter for ultrasound because they demonstrate how breast imaging AI enters real workflows: thresholds affect recall, sensitivity, specificity, arbitration workload, and positive predictive value. The same will be true for ultrasound AI. A high AUC is not enough; a system must improve the diagnostic pathway at a clinically acceptable false-negative rate.

Risk stratification determines whether supplemental ultrasound is worthwhile. A validated breast cancer risk model such as the Gail model is not an ultrasound tool, but it illustrates how imaging decisions can be personalized by baseline risk [49]. Dense breast tissue, family history, prior high-risk lesions, genetic predisposition, and previous chest irradiation all influence the choice of supplemental imaging. MRI is generally preferred for many high-risk patients when available, but ultrasound may be useful when MRI is contraindicated, unavailable, or as an adjunct for targeted evaluation. Computer-aided diagnosis in multiparametric MRI has been studied to reduce false-positive diagnoses in women with extremely dense breasts [50], again emphasizing that every modality faces the same balance between sensitivity and unnecessary workup.

Multimodal diagnosis is increasingly quantitative. Mammography AI can identify microcalcification malignancy risk [43], DBT radiomics can predict axillary lymph node metastasis [51], and DBT tissue matching can support comparison across ipsilateral tissue [44]. Ultrasound adds different information: real-time morphology, elasticity, vascularity, procedural accessibility, and dynamic lesion assessment. The future is unlikely to be a single winning modality. It is more likely to be a structured diagnostic system in which mammography/DBT, ultrasound, MRI, pathology, and clinical data each contribute task-specific information.

For AI implementation, ultrasound reports and image archives should be interoperable. Lesion location, size, clock-face position, distance from nipple, BI-RADS descriptors, acquisition plane, scanner information, biopsy recommendation, pathology, and follow-up should be stored in ways that support audit and model monitoring. Without this infrastructure, AI output remains a disconnected score rather than a dependable diagnostic tool.

## 10. AI for Axillary Staging, Lymphovascular Invasion, and Response Assessment

Axillary ultrasound is a familiar staging tool, but its accuracy is imperfect. AI models are being developed to quantify nodal morphology, texture, elasticity, vascularity, and tumor-node feature relationships. The goal is to improve preoperative risk estimation, guide biopsy targeting, and support selective axillary management.

Recent AI studies aim to improve this space with radiomics and deep learning. Shear-wave elastography radiomics has been used to assess axillary lymph node status in early-stage breast cancer [15]. CEUS radiomics has been studied for predicting axillary lymph node metastasis and prognosis [39]. Ultrasound-based deep learning radiomics in a multicenter study achieved strong discrimination for axillary lymph node metastasis and improved radiologists’ diagnostic accuracy [19]. Elastography-based AI has also been evaluated prospectively for predicting axillary status after neoadjuvant chemotherapy [16]. These studies target clinically meaningful endpoints because axillary management has moved away from routine extensive dissection toward selective surgery. Better preoperative and post-treatment nodal assessment could reduce morbidity if validated in outcome-based trials.

Lymphovascular invasion is another important prognostic feature. Ultrasound radiomics-based nomograms have been developed to predict lymphovascular invasion in invasive breast cancer [17,18]. The appeal is clear: LVI is associated with metastatic potential, recurrence risk, and treatment planning, but is traditionally known only after pathology. If ultrasound-based models can identify high-risk biology preoperatively, they could help tailor biopsy, MRI use, axillary sampling, and multidisciplinary discussion. Yet LVI prediction illustrates the boundary of imaging inference. A model may enrich probability, but it cannot provide the microscopic certainty of histopathology. The safest framing is risk stratification, not diagnosis.

Response assessment after neoadjuvant therapy is similarly complex. Ultrasound can measure tumor size and nodal morphology serially, but therapy-induced necrosis, fibrosis, fragmented residual disease, and nonmass patterns may reduce accuracy. Elastography, Doppler, CEUS, and radiomics may help by measuring stiffness, vascularity, and heterogeneity, but they should be integrated with MRI, clinical examination, pathology from clips or nodes, and tumor subtype. A practical future workflow may combine baseline ultrasound radiomics, interim response imaging, and pathology markers to estimate residual disease probability, but such workflows require prospective validation before changing surgery.

## 11. AI-Based Tumor Biology Prediction and Radiopathomics

One of the most active AI research directions is the use of ultrasound to infer tumor biology. Molecular subtype, HER2 status, HER2-low expression, Ki-67 expression, luminal versus non-luminal phenotype, and lymphovascular invasion are biologically meaningful because they influence prognosis and treatment. Ultrasound can potentially contribute because tumor biology affects growth pattern, stromal reaction, necrosis, vascularity, stiffness, and heterogeneity. Multimodal ultrasound studies have used assembled convolutional neural networks to decode molecular subtypes [20], and multicenter retrospective studies have used convolutional neural networks to assess molecular subtypes from ultrasound images [21]. A radiopathomics approach combining preoperative ultrasound images with biopsy whole-slide images distinguished luminal from non-luminal early-stage tumors [22]. Other multicenter studies have predicted Ki-67 expression [23], molecular subtype with interpretable imaging features [24], HER2 status from longitudinal and transverse ultrasound radiomics with clinical factors [25], and HER2-low breast cancer with ultrasound-based radiomics [26].

These studies are scientifically exciting because they move ultrasound beyond lesion detection toward precision medicine. A radiologist may already intuit that a round circumscribed high-grade cancer differs from a spiculated low-grade luminal cancer; radiomics formalizes that intuition and can extract patterns not visible to the eye. However, several constraints matter. First, biomarkers are measured on tissue and can vary across tumor regions. Second, ultrasound images are sensitive to machine vendor, preset, depth, gain, and acquisition plane. Third, many models are retrospective and may be affected by selection bias, class imbalance, or local treatment patterns. Fourth, clinical usefulness requires actionability. Predicting Ki-67 or HER2-low status is valuable only if it changes biopsy strategy, treatment selection, trial eligibility, or patient counseling.

Radiopathomics may be particularly promising because it acknowledges that imaging and pathology describe different levels of the same disease. Radiopathomics combines macroscopic imaging phenotype with microscopic tissue phenotype [22]. This approach could help identify sampling discordance, characterize intratumoral heterogeneity, or select lesions for additional biopsy. It also creates a more realistic path for AI: augmenting multidisciplinary diagnosis rather than producing a detached black-box label.

However, a critical limitation in AI-based biologic prediction—particularly in radiopathomics—is the inherent imperfection of the pathological “ground truth” itself. Training AI models to predict HER2-low status, Ki-67 expression, or molecular subtypes relies heavily on immunohistochemistry (IHC) labels. Yet, IHC is subject to well-documented inter-observer variability, differences in staining protocols, and subjective scoring by pathologists. Furthermore, the immense intratumoral heterogeneity of breast cancer means that a core needle biopsy may not accurately reflect the entire tumor biology, frequently leading to biopsy-versus-resection discordance. When an AI model’s output diverges from the initial biopsy result, it is not always the algorithm that has failed; the macroscopic ultrasound features may be capturing the heterogeneity of the entire mass that a focal needle sample missed. Therefore, future radiopathomics research must account for these ground-truth uncertainties and avoid treating single-site biopsy pathology as an infallible gold standard.

## 12. AI, Image-Guided Biopsy, and Imaging–Pathology Concordance

Ultrasound-guided biopsy remains the decisive diagnostic step when imaging is suspicious. The practical value of AI is greatest when model output is linked to procedural planning and imaging–pathology concordance.

The diagnostic process does not end when tissue is obtained. A benign pathology result after biopsy of a highly suspicious lesion remains discordant and should prompt repeat biopsy, vacuum-assisted sampling, surgical excision, or multidisciplinary review. Conversely, a benign concordant result after biopsy of a low-suspicion lesion may support follow-up. AI scores should be documented as supportive information, not as a reason to ignore discordance.

AI and radiomics do not remove this responsibility. In fact, they may increase the need for concordance discipline because models can create a false sense of precision. A model score should never be allowed to overrule a discordant clinical, imaging, or pathology picture without documented multidisciplinary review. The most mature clinical ultrasound systems will combine standardized reporting, biopsy tracking, pathology linkage, and audit of upgrades, downgrades, false negatives, and interval cancers.

## 13. Clinical Implementation and Governance of AI in Ultrasound

The evidence base for AI in ultrasound diagnosis is now broad enough to demand implementation discipline. The strongest near-term uses are those with clear endpoints and limited workflow disruption: second-reader support for mass classification, assistance for less experienced radiologists, structured triage of low-suspicion lesions, ABUS or video review support, and prioritization of suspicious findings for biopsy or expert review.

Clinical implementation of AI should start by defining the intended use. A tool built for diagnostic breast masses should not be used for screening whole-breast scans unless validated for that task. A model trained on still images should not be assumed to perform on video or automated volumes. A model validated in a tertiary center should not be assumed to perform in community clinics with different scanners, patient demographics, and disease prevalence. Prospective decision-support studies are especially valuable because they observe how radiologists behave with AI rather than merely comparing algorithm output with pathology [9,11,12]. Mammography AI experience reinforces the same lesson: different thresholds can shift recall, sensitivity, specificity, arbitration workload, and positive predictive value [45,46,47,48,52,53,54].

Beyond clinical performance, the transition of ultrasound AI from research to routine practice requires navigating complex regulatory pathways and technical infrastructures. In the United States and Europe, AI algorithms intended for clinical decision-making must undergo rigorous evaluation as Software as a Medical Device (SaMD), requiring FDA clearance or CE marking under the Medical Device Regulation (MDR). These regulatory frameworks increasingly demand evidence of algorithmic transparency, post-market surveillance, and generalizability across diverse demographics and scanner manufacturers.

To overcome the limitations of localized data without compromising patient privacy, Federated Learning (FL) has emerged as a crucial infrastructure. FL enables multiple institutions to train a shared global model collaboratively by exchanging encrypted model weights rather than raw ultrasound images. Furthermore, the paradigm of model architecture is rapidly shifting. While earlier models relied heavily on task-specific convolutional networks, future clinical deployment is moving toward Foundation Models and Vision Transformers (ViTs). These large-scale models, pre-trained on vast amounts of unannotated medical imaging data, can adapt to diverse downstream tasks—from mass segmentation to biologic prediction—with unprecedented robustness, representing the next frontier for AI-empowered ultrasound. Additionally, generative intelligence (such as Generative Adversarial Networks) is increasingly utilized for image synthesis and data augmentation to overcome dataset scarcity. However, integrating these advanced computer vision and deep learning algorithms requires robust computational intelligence, demanding specialized software-hardware tools such as high-performance Graphics Processing Units (GPUs) and scalable cloud-computing frameworks.

Reader behavior is central. AI can improve performance when it prompts reconsideration, but it can also create automation bias. A reader may accept a benign model output despite suspicious morphology, or biopsy a benign lesion because the model labels it high risk. Training should therefore include model failures, uncertainty thresholds, and rules for overriding AI when clinical or imaging findings are discordant.

AI safety and security also matter. A study of adversarial mammographic images showed that an AI diagnosis model could be fooled by image modifications and that human readers detected only a portion of adversarial samples [55]. Although that study used mammography, the lesson applies to ultrasound AI: medical models are software systems that require cybersecurity, version control, monitoring, and post-market surveillance. Scanner updates, compression algorithms, vendor changes, and population drift can degrade performance. A clinic deploying ultrasound AI should track not only aggregate AUC but also false-negative cases, cancers downgraded by AI, performance across age and breast density, scanner vendor, lesion type, and reader experience.

Equity is a further requirement for AI in ultrasound diagnosis. Ultrasound is often proposed as a solution for lower-resource environments because it is portable and less expensive than MRI. That promise will be undermined if AI tools are trained mainly on narrow populations or high-end scanners. The low-resource triage study of women with palpable lumps is important because it explicitly addresses a setting where access and workflow differ from academic hospitals [13]. Future ultrasound AI should report demographics, scanner vendors, acquisition protocols, disease prevalence, and subgroup performance as standard practice.

## 14. Limitations and Risks of AI in Ultrasound Diagnosis

AI-assisted ultrasound diagnosis inherits the limitations of ultrasound and adds new algorithmic risks. Ultrasound remains operator-dependent, has a limited field of view, and can miss lesions that are isoechoic, small, deep, masked by shadowing, or outside the scanned region. AI models can further fail through dataset bias, scanner drift, segmentation error, overfitting, poor calibration, and lack of external validation.

Reproducibility is a central problem. Radiomics features may change with scanner vendor, transducer frequency, gain, depth, compression, segmentation method, and preprocessing. Deep learning models can perform well in internal data but less well in external centers. Prospective testing, decision-curve analysis, subgroup reporting, and post-deployment monitoring are therefore as important as headline AUC. Furthermore, addressing these reproducibility challenges relies heavily on the expansion of publicly available ultrasound image datasets (such as the Breast Ultrasound Images Dataset [BUSI]). These open-access repositories are essential for providing standardized benchmarks to train, test, and compare deep learning and computer vision algorithms transparently across different research groups.

There is also a communication risk. Patients may hear “AI says benign” and interpret it as certainty. Clinicians may hear “radiomics predicts HER2-low” and confuse probability with tissue diagnosis. Reports and multidisciplinary discussions should use cautious language: “supports low probability,” “may help triage,” “requires pathologic confirmation,” and “should be interpreted with imaging–pathology concordance.” Good ultrasound diagnosis is not only image interpretation; it is risk communication.

## 15. Future Directions

The next stage of AI in ultrasound diagnosis will be multiparametric, standardized, and workflow-centered. B-mode images will remain essential, but elastography, Doppler, CEUS, automated volumes, video, clinical history, pathology, and prior imaging will increasingly be fused into task-specific models. The most successful tools will be those that reduce benign biopsy, support less experienced readers, improve axillary staging, identify patients needing additional imaging or sampling, and strengthen quality assurance.

Several developments are needed. First, acquisition protocols must be standardized enough for quantitative analysis while preserving the flexibility that makes ultrasound useful. Second, models should be externally validated across vendors, countries, clinical settings, and patient subgroups. Third, studies should move beyond enriched case–control datasets toward consecutive real-world cohorts. Fourth, outcome measures should include cancer detection, false-negative rate, benign biopsy rate, time to diagnosis, reader workload, patient anxiety, cost, and downstream treatment decisions. Fifth, AI systems should be integrated with reporting, image archiving, pathology, and follow-up databases so that performance can be audited continuously.

Multimodal integration will also become more important. The strongest diagnostic systems will combine mammography or DBT findings, ultrasound morphology, elasticity, vascularity, MRI when indicated, pathology, patient risk, and prior imaging. This is not merely a technical challenge. It requires clinical governance: deciding who sees the AI output, when it appears, how disagreements are handled, how upgrades and downgrades are documented, and how patients are informed. Breast ultrasound is well positioned for this future because it already sits at the interface of imaging, examination, procedure, and pathology.

## 16. Conclusions

Artificial intelligence is becoming an important diagnostic layer in ultrasound, particularly in breast imaging where recent multicenter evidence supports applications in lesion classification, BI-RADS decision support, elastography, Doppler and CEUS analysis, ABUS and video interpretation, axillary staging, and tumor biology prediction. The clinical goal is not autonomous replacement of radiologists or pathologists, but safer, more reproducible, and more efficient ultrasound diagnosis. AI should be implemented as decision support anchored in standardized acquisition, BI-RADS reporting, imaging–pathology concordance, external validation, cybersecurity, equity, and continuous audit. Used in this way, AI can help ultrasound move from operator-dependent image interpretation toward integrated, patient-centered diagnostic intelligence.

## Figures and Tables

**Table 1:** Major Clinical Applications of AI in Ultrasound Diagnosis.

AI-Supported Diagnostic Scenario	AI/Ultrasound Contribution	Typical Clinical Output	Implementation Cautions
Palpable mass or focal symptom	Real-time targeted imaging over the area of concern; correlation with mammography or DBT when age appropriate	Cystic versus solid characterization; BI-RADS assessment; biopsy target when suspicious	Negative imaging does not automatically dismiss a strongly suspicious clinical examination [4]
Mammographic, DBT, or MRI correlate	Clarifies whether an imaging finding has a sonographic correlate that can be biopsied under ultrasound guidance	Targeted lesion description, lesion size, distance from nipple/skin, biopsy feasibility	Calcifications and subtle distortions may remain better managed by mammographic or DBT guidance
Dense breast supplemental imaging	Adds radiation-free evaluation of dense tissue and may detect mammographically occult masses	Supplemental findings with BI-RADS assessment and follow-up or biopsy recommendation	Higher false-positive and short-term follow-up rates require shared decision-making [1]
BI-RADS 3 and 4A AI management	Supports short-interval surveillance or biopsy decisions; AI/CAD may provide second-reader probability estimates	Refined probability of malignancy; follow-up stability; procedural planning	AI downgrading requires prospective validation and preserved sensitivity [8,10,14]
Axillary staging	Evaluates lymph node morphology, cortex, hilum, vascularity, and biopsy target	Suspicious node identification; needle sampling; nodal burden estimation	Micrometastases and treatment response remain difficult; models must not replace pathology [15,16,19]
AI tumor phenotyping	Extracts radiomic and deep features from B-mode, elastography, Doppler, CEUS, automated-volume, or multimodal ultrasound	Exploratory prediction of subtype, HER2, Ki-67, LVI, and prognosis	Biologic prediction is promising but usually investigational [20,21,22,23,24,25,26]
Image-guided intervention	Enables real-time core biopsy, vacuum-assisted biopsy, cyst aspiration, clip placement, and localization	Tissue diagnosis and procedural documentation	Accurate imaging–pathology concordance remains mandatory

**Table 2:** Representative Recent Evidence for AI in Breast Ultrasound Diagnosis.

Representative Study			AI Application	Sample Size/Design	US Vendor Diversity	Headline Performance
Wei et al. (2022) [7]			Benign–malignant classification and BI-RADS decision support	901 patients/Prospective, multicenter	Multicenter	AUC: 0.906; Sens: 94.2%; NPV: 96.3%. Unnecessary biopsy rate decreased from 33.0% to 11.9%
Gu et al. (2023) [41]			Breast lesion risk stratification and BI-RADS categorization	5012 patients/Prospective, multicenter	32 hospitals (High diversity)	AUC: 0.980 (binary), 0.945 (BI-RADS); Sens: 90.9%; Spec: 78.7%
Cai et al. (2025) [38]			Shear wave elastography (SWE) for BI-RADS 3/4 evaluation	1294 patients/Retrospective and External Validation	12 institutions, 7 countries (International)	AUROC: 0.94; Sens: 97.9%; False-positive rate reduced by 62.1%
Zhao et al. (2022) [14]	Malignancy rate reduction among BI-RADS 4A lesions	479 BI-RADS 4A lesions/Retrospective	Not specified		AUROC: 0.897; Sens: 92.6%; NPV: 78.4%	
Zhang et al. (2025) [19]			Axillary lymph node metastasis (ALNM) assessment	866 patients/Multicenter with prospective test set	6 hospitals	AUC: 0.95 (prospective). Radiologist AUC improved from 0.75 to 0.85 (senior)

## Data Availability

No new data were created or analyzed in this study. Data sharing is not applicable to this article.
